# Bilateral extensive multiple multi-layered retinal haemorrhage in a patient with alcoholic liver disease

**DOI:** 10.11604/pamj.2018.29.89.10869

**Published:** 2018-01-30

**Authors:** Ibrahim Elaraoud, Erica Damato

**Affiliations:** 1 Birmingham Midland Eye Centre, Birmingham, UK

**Keywords:** Retinal haemorrhages, liver disease, INR

## Image in medicine

We report a rare case of bilateral multiple bilateral vitreous, pre-retinal and intra-retinal haemorrhages after bouts of coughing in a 47 year old female patient with high international normalized ratio (INR) due to alcoholic liver disease. Patient has been complaining of 2 weeks history of reduced vision to her liver physician who referred her to local eye unit. She reported that the vision deteriorated following intense coughing. She was found to have bilateral vitreous, pre-retinal and intra-retinal haemorrhages. liver disease. The patient was kept under ophthalmic observation. The vitreous, preretinal and intraretinal haemorrhages resolved spontaneously and his vision recovered fully Alcoholic liver disease is becoming more common. Clinicians should be aware that the combination of increased intravenous pressure due to persistent coughing and possible early portal hypertension and a pathological increase in INR can lead to severe multiple multi-layered retinal haemorrhage. The haemorrhages, however, will resolve spontaneously in most cases. In foveal involving pre-retinal haemorrhage, YAG- laser membranotomy was shown to speed up visual recovery in previous reports.

**Figure 1:**
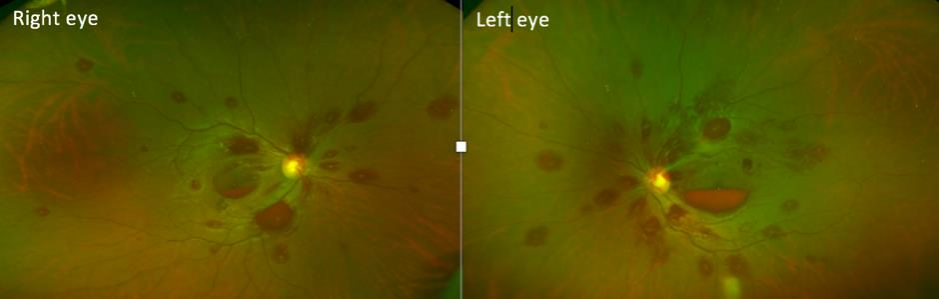
bilateral retinae, with multi-leveled retinal haemorrhage

